# Supplementary material for: Lactoferrin Induces Erythropoietin Synthesis and Rescues Cognitive Functions in the Offspring of Rats Subjected to Prenatal Hypoxia
Source: Nutrients. 2022 Mar 27;14(7):1399. doi: 10.3390/nu14071399 (PMC9003537; doi:10.3390/nu14071399)

Figure S1. EPO detection by Western blotting in various tissues of embryos from rats treated differently. # - number of corresponding lanes.

Groups		Analyzed part of pregnant rats or fetus			
		Brain of pregnant females	Placenta	Brain of the embryos	Torso of the embryos
Embryos (*n* = 10) from control pregnant rats (*n* = 2) with *i.p.* saline injections during gestation	EPO presence	0	0	0	0
	EPO absence	**2** (##1, 2)	**10** (##82, 83, 84, 85, 91, 92, 93, 94, 95, 96)	**10** (## 73, 74, 75, 76, 77, 78, 79, 86, 87, 88)	**10** (## 64, 65, 66, 67, 68, 69, 70, 71, 98, 99)
Embryos (*n* = 16) from pregnant rats (*n* = 2) with hypoxia and *i.p.* saline injections during gestation	EPO presence	0	0	0	0
	EPO absence	**2** (##3, 101)	**16** (## 19, 20, 21, 46, 47, 48, 49, 50, 51, 52, 97, 102, 103, 104, 105, 106)	**16** (## 22, 23, 24, 37, 38, 39, 40, 41, 42, 43, 55, 56, 57, 58, 59, 60)	**16** (## 10, 11, 12, 13, 14, 15, 25, 28, 29, 30, 31, 32, 33, 34, 61, 62)
Embryos (*n* = 12) from pregnant rats (*n* = 2) with hypoxia and *i.p.* apo-rhLF injections during gestation	EPO presence	**2** (## 4, 5)	0	**12** (## 6, 7, 8, 9, 16, 17, 18, 35, 36, 44, 45, 53)	**12** (## 26, 27, 54, 63, 72, 80, 81, 89, 90)
	EPO absence	0	**12** (##107, 108, 109, 110, 111, 112, 113, 114, 115, 116, 117, 118)	0	0

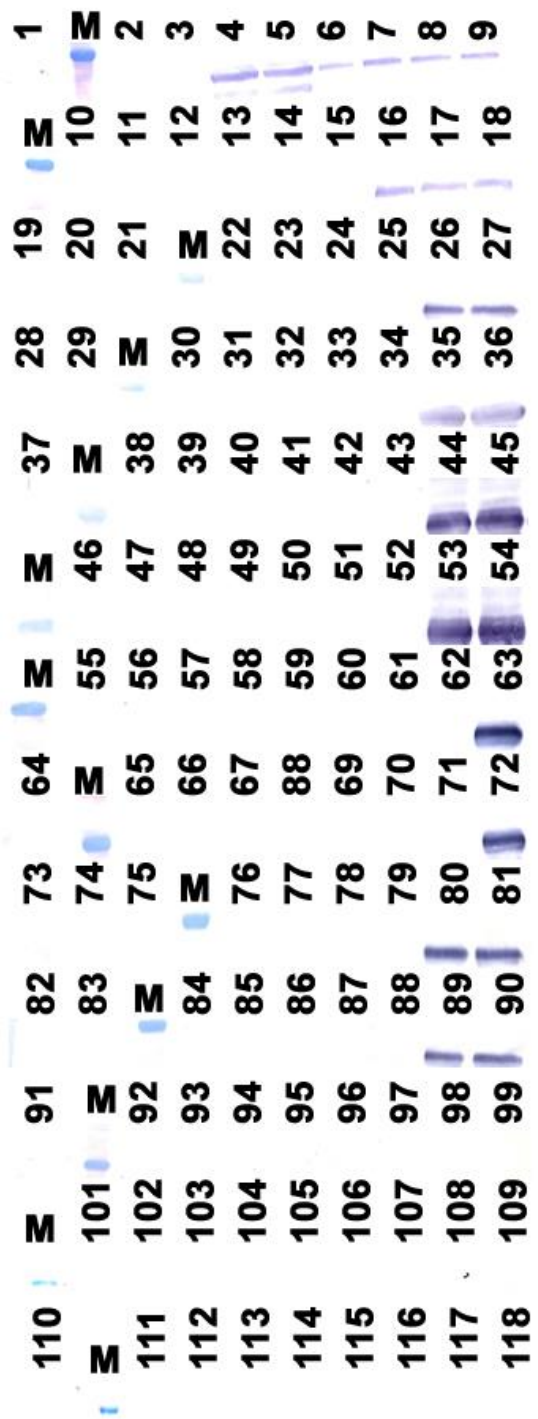

Supplement: Supplementary file 1 [file nutrients-14-01399-s001.zip › nutrients-1538649-supplementary.pdf]
